# Supplementary material for: Clinical Presentations of Adolescents Aged 16–18 Years in the Adult Emergency Department
Source: Int J Environ Res Public Health. 2021 Sep 11;18(18):9578. doi: 10.3390/ijerph18189578 (PMC8470799; doi:10.3390/ijerph18189578)
Supplement: Supplementary file 1 [file ijerph-18-09578-s001.zip › Sup. Tab.S2.pdf]

Supplement Table S2. Demographics, type of ED use, principle complaints of adolescents aged 16-18 year compared to adults ≥25-35 years.

**Category**	**≥16-18 n=4,930**		**≥25-35 n=35,611**		**p**
**Age, med (IQR)**	17	(16-17)	29	(27-32)	**<0.001**
**Gender female, n (%)**	2,543	(51.6)	16,292	(45.8)	**<0.001**
**Year, n (%)**					**<0.001**
2013	1,009	(20.5)	6,319	(17.7)	
2014	919	(18.6)	6,725	(18.9)	
2015	873	(17.7)	7,236	(20.3)	
2016	1,061	(21.5)	7,628	(21.4)	
2017	1,068	(21.7)	7,703	(21.6)	
**Presentation Date and Time, n (%)**					
Saturday or Sunday admission (00:00-23:59), n (%)	1,497	(30.4)	10,996	(30.9)	0.465
Night-time admissions (19:00 Ð 06:59), n (%)	1,929	(39.1)	12,969	(36.4)	**<0.001**
Public and cantonal (Bern) holidays, n (%)	98	(2.0)	743	(2.1)	0.649
**Day of the week, n (%)**					0.551
Monday	712	(14.4)	5,278	(14.8)	
Tuesday	666	(13.5)	4,776	(13.4)	
Wednesday	675	(13.7)	4,726	(13.3)	
Thursday	663	(13.4)	4,884	(13.7)	
Friday	717	(14.5)	4,951	(13.9)	
Saturday	737	(14.9)	5,632	(15.8)	
Sunday	760	(15.4)	5,364	(15.1)	
**Type of admission, n (%)**					**<0.001**
Ambulance	506	(10.3)	2,989	(8.4)	
General Practitioner	170	(3.4)	1,036	(2.9)	
External Hospital	243	(4.9)	1,406	(3.9)	
Police	73	(1.5)	712	(2.0)	
Air Rescue	65	(1.3)	264	(0.7)	
Repatriation	5	(0.1)	34	(0.1)	
Walk-In	2,674	(54.2)	19,942	(56.0)	
Internal Referral	180	(3.7)	1,482	(4.2)	
Emergency care centre/doctor	49	(1.0)	394	(1.1)	
Other	12	(0.2)	128	(0.4)	
Missing Information	953	(19.3)	7,224	(20.3)	
**Triage, n (%)**					0.191
Life-threatening	148	(3.0)	1,025	(2.9)	
Highly urgent	889	(18.0)	6,015	(16.9)	
Urgent	3,217	(65.3)	23,869	(67.0)	
Semi-urgent	448	(9.1)	3,074	(8.6)	
Non-urgent	125	(2.5)	842	(2.4)	

Missing Information	103	(2.1)	786	(2.2)	
**Resuscitation room, n (%)**					**<0.001**
No	4,704	(95.4)	34,397	(96.6)	
Yes	226	(4.6)	1,214	(3.4)	
**Principle complaints, n (%)**					**<0.001**
Psychiatric problem, including self-harm	466	(9.5)	3,215	(9.0)	
Musculoskeletal problems including rheumatological problems	677	(13.7)	4,995	(14.0)	
Gastrointestinal problems	75	(1.5)	335	(0.9)	
Respiratory problems	127	(2.6)	837	(2.4)	
Neurological problems	349	(7.1)	2,967	(8.3)	
Cardiovascular problems	53	(1.1)	968	(2.7)	
Infectious disease, including skin problems	353	(7.2)	2,692	(7.6)	
Obstetric or gynaecological problems	0	(0.0)	10	(0.0)	
Dental problems	12	(0.2)	120	(0.3)	
Eye problems	272	(5.5)	2,438	(6.8)	
Other	306	(6.2)	2,783	(7.8)	
Trauma	1,200	(24.3)	6,714	(18.9)	
Genitourinary problems	125	(2.5)	1,176	(3.3)	
Ear/Nose/Throat problems	371	(7.5)	2,443	(6.9)	
Follow Up	132	(2.7)	926	(2.6)	
Missing Information	412	(8.4)	2,992	(8.4)	
**Discharge, n (%)**					**0.009**
Death	0	(0.0)	16	(0.0)	
Discharge home	3,490	(70.8)	24,246	(68.1)	
Hospital admission	562	(11.4)	4,353	(12.2)	
Transfer to external hospital	320	(6.5)	2,588	(7.3)	
Other	44	(0.9)	364	(1.0)	
Not specified	514	(10.4)	4,042	(11.4)	
Missing Information	0	(0.0)	2	(0.0)	
