# Supplementary material for: Clinical Presentations of Adolescents Aged 16–18 Years in the Adult Emergency Department
Source: Int J Environ Res Public Health. 2021 Sep 11;18(18):9578. doi: 10.3390/ijerph18189578 (PMC8470799; doi:10.3390/ijerph18189578)
Supplement: Supplementary file 1 [file ijerph-18-09578-s001.zip › Sup. Tab.S3.pdf]

Supplement Table S3. Demograhics, type of ED use, principle complaints of young adults ≥18-25 years compared to adults ≥25-35 years.

**Category**	**≥18-25 n=23,221**		**≥25-35 n=35,611**		**p**
**Age, med (IQR)**	21	(19-23)	29	(27-32)	**<0.001**
**Gender female, n (%)**	10,944	(47.1)	16,292	(45.8)	**<0.001**
**Year, n (%)**					0.811
2013	4,167	(17.9)	6,319	(17.7)	
2014	4,427	(19.1)	6,725	(18.9)	
2015	4,656	(20.1)	7,236	(20.3)	
2016	5,006	(21.6)	7,628	(21.4)	
2017	4,965	(21.4)	7,703	(21.6)	
**Presentation Date and Time, n (%)**					
Saturday or Sunday admission (00:00-23:59), n (%)	7,314	(31.5)	10,996	(30.9)	0.113
Night-time admissions (19:00 Ð 06:59), n (%)	9,112	(39.2)	12,969	(36.4)	**<0.001**
Public and cantonal (Bern) holidays, n (%)	474	(2.0)	743	(2.1)	0.707
**Day of the week, n (%)**					0.052
Monday	3,346	(14.4)	5,278	(14.8)	
Tuesday	3,084	(13.3)	4,776	(13.4)	
Wednesday	3,103	(13.4)	4,726	(13.3)	
Thursday	3,146	(13.5)	4,884	(13.7)	
Friday	3,228	(13.9)	4,951	(13.9)	
Saturday	3,585	(15.4)	5,632	(15.8)	
Sunday	3,729	(16.1)	5,364	(15.1)	
**Type of admission, n (%)**					**<0.001**
Ambulance	2,147	(9.2)	2,989	(8.4)	
General Practitioner	638	(2.7)	1,036	(2.9)	
External Hospital	926	(4.0)	1,406	(3.9)	
Police	330	(1.4)	712	(2.0)	
Air Rescue	229	(1.0)	264	(0.7)	
Repatriation	18	(0.1)	34	(0.1)	
Walk-In	12,972	(55.9)	19,942	(56.0)	
Internal Referral	904	(3.9)	1,482	(4.2)	
Emergency care centre/doctor	284	(1.2)	394	(1.1)	
Other	49	(0.2)	128	(0.4)	
Missing Information	4,724	(20.3)	7,224	(20.3)	
**Triage, n (%)**					0.421
Life-threatening	629	(2.7)	1,025	(2.9)	
Highly urgent	3,818	(16.4)	6,015	(16.9)	
Urgent	15,638	(67.3)	23,869	(67.0)	
Semi-urgent	2,074	(8.9)	3,074	(8.6)	
Non-urgent	558	(2.4)	842	(2.4)	

Missing Information	504	(2.2)	786	(2.2)	
**Resuscitation room, n (%)**					0.105
No	22,370	(96.3)	34,397	(96.6)	
Yes	850	(3.7)	1,214	(3.4)	
**Principle complaint, n (%)**					**<0.001**
Psychiatric problem, including self-harm	2,275	(9.8)	3,215	(9.0)	
Musculoskeletal problems including rheumatological problems	3,023	(13.0)	4,995	(14.0)	
Gastrointestinal problems	282	(1.2)	335	(0.9)	
Respiratory problems	502	(2.2)	837	(2.4)	
Neurological problems	1,752	(7.5)	2,967	(8.3)	
Cardiovascular problems	437	(1.9)	968	(2.7)	
Infectious disease, including skin problems	1,560	(6.7)	2,692	(7.6)	
Obstetric or gynaecological problems	1	(0.0)	10	(0.0)	
Dental problems	96	(0.4)	120	(0.3)	
Eye problems	1,538	(6.6)	2,438	(6.8)	
Other	1,691	(7.3)	2,783	(7.8)	
Trauma	5,147	(22.2)	6,714	(18.9)	
Genitourinary problems	807	(3.5)	1,176	(3.3)	
Ear/Nose/Throat problems	1,608	(6.9)	2,443	(6.9)	
Follow Up	571	(2.5)	926	(2.6)	
Missing Information	1,931	(8.3)	2,992	(8.4)	
**Discharge, n (%)**					0.061
Death	5	(0.0)	16	(0.0)	
Discharge home	16,056	(69.1)	24,246	(68.1)	
Hospital admission	2,678	(11.5)	4,353	(12.2)	
Transfer to external hospital	1,631	(7.0)	2,588	(7.3)	
Other	222	(1.0)	364	(1.0)	
Not specified	2,627	(11.3)	4,042	(11.4)	
Missing Information	2	(0.0)	2	(0.0)	
